# Dementia in Rare Genetic Neurodevelopmental Disorders: A Systematic Literature Review

**DOI:** 10.1212/WNL.0000000000209413

**Published:** 2024-05-17

**Authors:** Hadassa Kwetsie, Malu van Schaijk, Sven Van Der Lee, Dederieke Maes-Festen, Leontine W. Ten Hoopen, Mieke M. van Haelst, Michael Coesmans, Esther Van Den Berg, Marie Claire Y. De Wit, Yolande Pijnenburg, Eleonora Aronica, Erik Boot, Agnies M. Van Eeghen

**Affiliations:** From Emma's Children's Hospital (H.K., A.M.V.E.), University of Amsterdam; Advisium (H.K., E.B., A.M.V.E.), 's Heeren Loo Zorggroep, Amersfoort; Department on Aging (M.S.), Netherlands Institute of Mental Health and Addiction (Trimbos Institute), Utrecht; Alzheimer Center Amsterdam (S.V.D.L., Y.P.), Amsterdam University Medical Center; Section Genomics of Neurodegenerative Diseases and Aging (S.V.D.L.), Department of Human Genetics Amsterdam UMC; Intellectual Disability Medicine (D.M.-F.), Department of General Practice, Erasmus MC, University Medical Center Rotterdam; ENCORE Expertise Center for Neurocognitive Disorders and Department of Pediatric Neurology (L.W.T.H., M.C.Y.D.W.), Sophia Children's Hospital, Erasmus MC University Medical Center Rotterdam; Erasmus School of Health Policy & Management (L.W.T.H.), Erasmus University Rotterdam; Department of Clinical Genetics (M.M.H.); Department of Human Genetics (M.M.H.), Amsterdam UMC, University of Amsterdam; Emma Center for Personalized Medicine (M.M.H., A.M.V.E.), Amsterdam University Medical Centers; Department of Psychiatry, Erasmus MC University Medical Center, Rotterdam; Department of Neurology and Alzheimer Center Erasmus MC (E.V.D.B.), Erasmus MC University Medical Center, Rotterdam; Amsterdam Neuroscience (Y.P.), Neurodegeneration; Department of (Neuro)Pathology, Amsterdam Neuroscience (E.A.), Amsterdam UMC, University of Amsterdam; Stichting Epilepsie Instellingen Nederland (SEIN) (E.A.), Heemstede, The Netherlands; The Dalglish Family 22q Clinic (E.B.), University Health Network, Toronto, Canada; and Department of Psychiatry and Neuropsychology (E.B.), Maastricht University, Maastricht University, The Netherlands.

## Abstract

**Background and Objectives:**

Knowledge of young-onset Alzheimer disease in adults with Down syndrome has greatly improved clinical care. However, little is known about dementia in rare genetic neurodevelopmental disorders (RGNDs). In this review, a comprehensive overview is provided of reports on dementia and cognitive/adaptive trajectories in adults with RGNDs.

**Methods:**

A systematic literature review was conducted in Embase, Medline ALL, and PsycINFO on December 6, 2022. The protocol was registered in PROSPERO (CRD42021223041). Search terms for dementia, cognitive and adaptive functioning, and RGNDs were combined using generic terms and the Orphanet database. Study characteristics and descriptive data on genetic diagnosis, clinical and neuropathologic features, comorbidities, and diagnostic methods were extracted using a modified version of the Cochrane Data Extraction Template.

**Results:**

The literature search yielded 40 publications (17 cohorts, 23 case studies) describing dementia and/or cognitive or adaptive trajectories in adults with 14 different RGNDs. Dementia was reported in 49 individuals (5 cohorts, 20 cases) with a mean age at onset of 44.4 years. Diagnostics were not disclosed for half of the reported individuals (n = 25/49, 51.0%). A total of 44 different psychodiagnostic instruments were used. MRI was the most reported additional investigation (n = 12/49, 24.5%). Comorbid disorders most frequently associated with cognitive/adaptive decline were epilepsy, psychotic disorders, and movement disorders.

**Discussion:**

Currently available literature shows limited information on aging in RGNDs, with relatively many reports of young-onset dementia. Longitudinal data may provide insights into converging neurodevelopmental degenerative pathways. We provide recommendations to optimize dementia screening, diagnosis, and research.

## Introduction

Rare genetic neurodevelopmental disorders (RGNDs) are characterized by a variety of neurologic and psychiatric symptoms, often involving intellectual disability (ID) and somatic comorbidity in various organ systems. By the European definition, diseases are rare when affecting fewer than 1 in 2,000.^1^ Although individually rare, together RGNDs are estimated to affect 1%–3% of the total population and millions of people globally.^1^ Improvement of health care has resulted in increasing life expectancy for this patient population, revealing age-related diseases such as dementia.

It is well-known that Down syndrome (DS), a common cause of ID, is associated with young-onset Alzheimer dementia due to the triplication of the amyloid precursor protein gene located on chromosome 21. This knowledge has resulted in targeted screening and diagnostic recommendations,^2^ greatly improving overall care for adults with DS. In the general population with ID (not caused by DS), varying prevalence rates of dementia have been reported, ranging from rates similar to the general population^3-5^ to rates up to 4 times higher,^6-14^ with both late^3,4^ and young onset (younger than 65 years).^11,14^ Higher dementia prevalences with young onset would be plausible in ID due to a lower premorbid cognitive reserve.^15^ In addition to genetic risk factors, comorbid disorders in RGNDs may also contribute to accelerated cognitive decline. Epilepsy, for instance, occurs in 20%–30% of individuals with ID, and age at onset, seizure type, duration of disease, and chronic use of antiseizure medication (ASM) have been associated with cognitive decline.^16^ Other common comorbidities associated with cognitive decline include psychosis, schizophrenia,^17^ and mood disorders.^18^ Diagnostic manuals state that to meet dementia criteria, cognitive decline cannot be *better explained by another mental disorder*. In patients with RGNDs, diagnosis may be more challenging because (reversible) psychiatric disorders may be causative or co-occurring and difficult to distinguish from dementia. Other diagnostic challenges in those with RGNDs include limited availability and validity of neuropsychological instruments,^19^ invasiveness of additional medical investigations, and limited guidance for cognitive monitoring of genetic conditions other than DS.

Hence, many questions remain regarding epidemiology, phenomenology, determinants, and mechanisms of dementia in RGNDs. This knowledge is necessary to improve care by identifying care gaps and targets for prevention, screening, diagnosis, monitoring, and treatment. In this study, we aim to provide a systematic review of knowledge on dementia, cognitive/adaptive trajectories, and associated factors in adults with RGNDs.

## Methods

**Figure 1:**
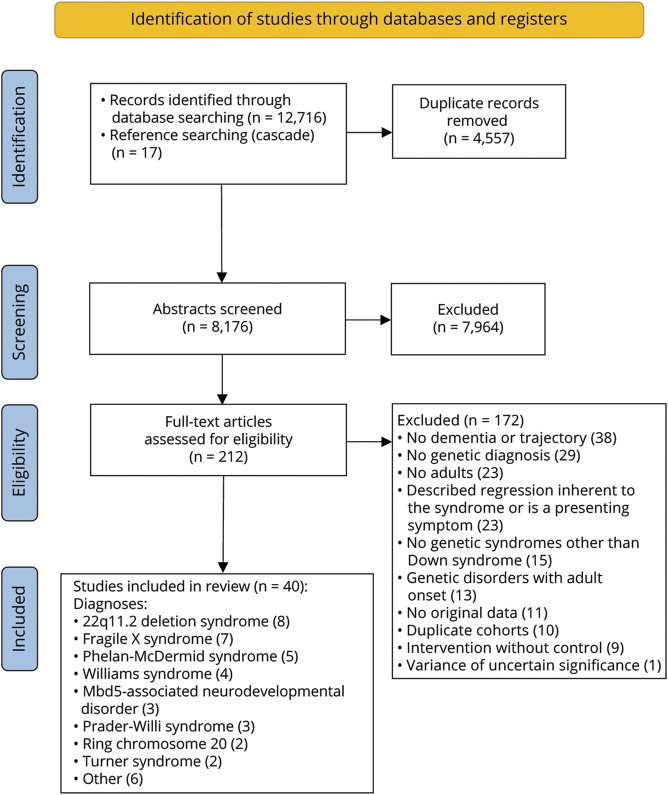
PRISMA Flowchart

We followed and used the template of the Preferred Reporting Items for Systematic Reviews and Meta-Analysis (PRISMA) checklist (Figure 1).^20^ The methodologic framework was published in advance in PROSPERO International Prospective Register of Systematic Reviews (CRD42021223041).

### Eligibility

Inclusion criteria consisted of all peer-reviewed studies in adults with molecularly confirmed RGNDs that reported on dementia and/or cognitive/adaptive functioning. Cognitive functioning is defined as the performance of mental processes such as memory and perception, whereas adaptive functioning refers to everyday tasks required for a person to fulfill typical roles in society, such as self-help, domestic skills, and communication.^21^ Dementia was defined as a significant decline from a prior level of cognitive and adaptive functioning. Studies reporting on cognitive/adaptive functioning required a longitudinal design or cross-sectional design that reported results for different age groups. Trajectories spanning both childhood and adulthood were excluded when participants were underaged during the majority of the interval. We defined RGNDs according to the Orphanet database as rare disorders with a genetic etiology affecting the nervous system in early development.^22^ Genetic disorders with adult onset, gene variants of uncertain significance, and Down syndrome (prevalence > 1:2000) were excluded. To target aging rather than developmental processes, we also excluded RGNDs in which cognitive decline is a presenting symptom, such as childhood dementias and neurometabolic disorders (see eTable 1 for an overview of excluded disorders).

### Search Strategy, Study Selection, Risk of Bias, and Quality Assessment

A literature search was performed in Embase, Medline ALL (Ovid), and PsycINFO (Ovid) on December 6, 2022, with the assistance of clinical research librarians (J.D. and M.F.M.E.). The search included (1) terms on dementia and cognitive or adaptive functioning and (2) terms regarding RGNDs including all rare genetic and chromosomal disorders from the Genetic and Rare Disease Information Center of the NIH (search terms in eTable 2). Additional articles were identified by citation tracking (n = 17). Authors were approached for adult-specific data in 8 cohorts that included both children and adults, which was provided by 1 author (acknowledgements). Three adults in 2 children cohorts were included as case studies because information on their cognitive trajectories was provided.

Rayyan, an application for systematic reviews, was used for title and abstract screening.^23^ All titles and abstracts were screened for relevance by 4 reviewers (A.M.v.E., L.t.H., M.v.S., and H.K.). A 10% subsample was screened for interrater reliability using the Cohen Kappa statistic to determine consistency between raters. Full-text articles were screened by 2 reviewers, where data were extracted and abstracted by one reviewer and checked by a second reviewer (M.v.S., H.K.). Discrepancies were discussed until consensus was reached. For quality appraisal, the Newcastle-Ottawa scale that allows the appraisal of the methodologic qualities of nonrandomized studies was used.^24^

### Data Extraction and Abstraction

**Figure 2:**
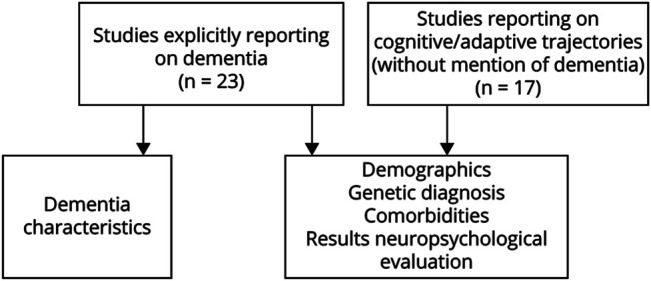
Overview of Study Selection and Data Extraction

We extracted study characteristics and descriptive data using a modified version of the Cochrane Consumers and Communication Review Group's Data Extraction Template.^25^ Data were extracted and abstracted on general information (first author, year of publication, and study design), patient characteristics (age, sex, level of ID, and premorbid intelligence quotient), genetics (genetic diagnosis, relevant coexisting diagnoses, and molecular test results), cognitive/adaptive trajectories, dementia features (prevalence, age at onset, etiology, neuropathology, and diagnostics), medication, epilepsy characteristics (seizure type and age at onset), other comorbidity, and use of psychodiagnostic instruments (Figure 2).

### Standard Protocol Approvals, Registrations, and Patient Consents

### Data Availability

Any data not published within the manuscript will be shared upon request.

## Results

Of 12,733 identified citations, 40 studies met the inclusion criteria (see eReferences e1–e40 and eTables 3–4), describing dementia (n = 23, 57.5%) or cognitive or adaptive trajectories without dementia diagnosis (n = 17, 42.5%) in adults with RGNDs (flowchart Figure 1) (view eTables 5 and 6 for all included trajectories, including stable or improving). A total of 17 cohorts (6 prospective, 7 retrospective, and 4 cross-sectional) and 23 case studies were identified, reporting on a total of 3,089 individuals with 14 different genetically confirmed RGNDs. The cohorts comprised 12 convenience samples from care facilities or (inter)national consortia, 2 population-based studies, 2 retrospective chart reviews, and 1 voluntarily sample from an online community.

### Individuals

#### Cohorts

In 17 cohorts, a total of 3,056 individuals were included, of whom 1,496 were female (49.0%, excluding 1 cohort in which sex was unknown). The average age at last assessment was 33.7 (range 19–52) years. At baseline, a mean full-scale IQ (FSIQ) score was reported in 13 cohorts with a mean of 64.6 (range 43.7–77.2).

#### Case Studies

In 23 case studies, a total of 32 individuals were described, of whom 17 were female (53.1%, sex of 1 case unknown). The average age was 46.5 (range 18–77) years. ID was absent in 6 cases (18.8%), unspecified in 6 (18.8%), and confirmed in 20 (62.5%), further specified in 18 as borderline (n = 2), mild (n = 7), moderate (n = 5), and severe ID (n = 4). At baseline, FSIQ was reported in 9 cases with a mean of 70.2 (range 38–114) and a developmental age in 6 cases with a mean of 5.6 (range 3–9) years, corresponding to moderate ID. No family histories of dementia were reported, aside from relatives affected by the same RGND.^e14,e18,e24,e25^

### Dementia in RGNDs

#### Prevalence

Dementia was reported in 5/17 (29.4%) cohort studies and in 20/32 (62.5%) separate case reports, describing a total of 49 individuals with 12 different RGNDs (Table 1). Two possible cases with dementia and 1 probable case with dementia were reported in a small population-based cohort of Prader-Willi syndrome (n = 3, 16.7%),^e40^ based on neuropsychological assessments. In a 22q11.2 deletion syndrome cohort from a psychiatric outpatient clinic,^e9^ 3 individuals had dementia (n = 3, 9.1%) (shown as separate cases) and 18 others showed both cognitive and adaptive decline without a formal diagnosis of dementia (n = 18, 54.5%). Dementia was also reported in small cohorts with Williams syndrome (n = 3, 13.6%)^e28^ and Dravet syndrome (n = 1, 4.5%) from care facilities,^e7^ but the certainty and method of diagnoses were not discussed. Medical records of a sample of fragile X syndrome,^e33^ referred for clinical or research evaluations, reported *cognitive decline or dementia* (n = 6, 9.7%), but no further details were provided, and it is unclear whether all individuals had been screened. In a national registry–based cohort of 1,349 individuals with neurofibromatosis type 1,^e17^ medical records were screened for *dementia-related encounters*, such as a dementia-related hospital visit, ICD-10 code, or purchase of antidementia drugs. A prevalence of 1.2% dementia-related encounters was reported, with increased risks of dementia (HR = 1.67), Alzheimer disease (AD) (HR = 2.88), and dementia-related death (RR = 2.42).

**Table 1:** Reports of Dementia in Rare Genetic Neurodevelopmental Disorders

RGND	Dementia prevalence	Sex f/m	(Mean) Age at onset	(Mean) Age at diagnosis	Diagnostic method	Diagnostic results of cases with dementia	Terminology as per the author	Suggested dementia etiology
Cohort studies reporting on dementia (5)								
Prader-Willi syndrome	3/18 16.6%	3/0	53, 40, 40	55, 45, 41	NPA	CAMDEX-DS impaired (1), WAIS decline (1), clinical impression only (1)	Dementia	Alzheimer disease
Williams syndrome	3/22 13.6%	n/a	n/a	n/a	Unknown	n/a	Dementia	Vascular dementia
Fragile X syndrome	6/62 9.7%	n/a	n/a	n/a	Unknown	n/a	Cognitive decline or dementia	n/a
Dravet syndrome	1/22 4.5%	0/1	n/a	55	NPA, MRI, EEG, autopsy	Cerebellar atrophy, periventricular white matter loss, myelin loss in medulla and cervical spinal cord	Dementia	Epileptic encephalo-pathy
Neurofibromatosis type 1	16/1.349 1.2%	8/8	n/a	74.2 (9.0)^b^	Unknown	n/a	Dementia	Alzheimer disease, vascular dementia, other
Case studies reporting on dementia (20)								
RGND	Sex	Age at onset	Age at diagnosis	Areas of decline	Diagnostic method	Diagnostic results	Terminology as per the author	Suggested dementia etiology
22q11.2 deletion syndrome	M	36	52	Memory, orientation, behavior, speech, adaptive function	NPA	8 y: IQ 75-80 36 y: IQ <45 44 y: IQ 26 52 y: IQ 21	Dementia	n/a
	M	n/a	38	Cognition, adaptive function	NPA	18 y: mild ID 29 y: mild/moderate ID 36 y: severe ID	Dementia	n/a
	F	23	29	Cognition, adaptive function, social skills	NPA	22 y: DA 6.4 25 y: mild ID 28 y: DA 4.8 30 y: DA 2.4	Dementia	n/a
3q29 deletion syndrome	F	56	57	Language, memory	NPA	56 y: FSIQ 62 MMSE 23/30 MoCA 11/30	Dementia	n/a
Cardiofaciocutaneous syndrome	M	Early 30s	39	Memory, EF, speech, adaptive function, motor	NPA, CT	15 y: WRAT2 3/3 38 y: BNA-R results all impaired	Dementia	n/a
Fragile X syndrome	M	69	77	Cognition, motor	NPA, MRI, autopsy	60 y: FSIQ 67 71 y: MMSE 25/30 74 y: MMSE 22/30	Progressive neurodegenerative syndrome	FXTAS, Parkinson disease dementia, Alzheimer disease
	M	64	70	Cognition, EF, disorientation, motor, mood, sleep	NPA, MRI	68 y: MMSE 10/30	Late onset neurologic symptoms consistent with the diagnosis of FXTAS	FXTAS
	M	Late 50s	65	Memory, orientation, motor	NPA, MRI	53 y: FSIQ 71 65 y: FSIQ 52 MMSE 7/30 severe global atrophy, ventriculomegaly, and WM intensities	Significant dementia	FXTAS
	M	71	77	Cognition, motor	NPA, MRI	60 y: FSIQ 67 71 y: MMSE 25/30 subcortical WM disease and dilated ventricles 77 y: MMSE 13/30	Progressive cognitive decline/neurodegeneration	FXTAS
Mbd5-associated neurodevelopmental disorder	F	n/a	60	Cognition	NPA, MRI	36 y: DA 9; 0 56 y: DA 5; 1–6; 11 60 y: DA 3; 8-5; 3 pronounced folia of the cerebellar vermis	Early-onset dementia	n/a
	F	46	48	Cognition, behavior, speech	NPA, MRI, LP, FDG-PET, blood tests	46 y: MMSE 29/30 48 y: Bilateral frontotemporal and parietal lobe hypometabolism in FDG-PET, MRI, and CSF unremarkable	Mild dementia	n/a
	M	Unknown	44^a^	Cognition, behavior	n/a	n/a	Early-onset dementia	n/a
Phelan-McDermid syndrome	F	30	33	Adaptive function, motor, speech	MRI	No abnormalities in MRI	Progressive process in the CNS/early debut of dementia	n/a
	M	16	18	Mood, motor, attention, speech	MRI, LP, blood tests	Low amyloid beta (479 mg/L), low total tau (82 ng/L), normal phosphorylated tau (24 mg/L) protein in LP	Early dementia	Alzheimer disease
	F	43	47^a^	Motor, cognition	MRI	n/a	Severe progressive neuro-degeneration	n/a
	F	39	40^a^	Motor, speech	MRI	n/a	Severe progressive neuro-degeneration	n/a
Prader-Willi syndrome	F	n/a	72	Memory, adaptive behavior	NPA	56 y:DA 6; 6 59 y: DA 4; 4	Early dementia	n/a
	F	40	58	Cognition, motor, adaptive behavior	NPA, CT	53 y: DA 1; 2–4; 10, SRZ total 5 58 y: DA 0; 8-1; 9, SRZ total 3, DSDS total 33	Dementia	n/a
Tuberous sclerosis complex	M	n/a	52	Behavior, mood, language, EF, visual episodic memory, processing speed	NPA, MRI	Atrophy and white matter lesions in temporal and frontal lobes	Dementia	Behavioral variant frontotemporal dementia
Turner syndrome	F	n/a	59	Memory, orientation	Tomography, X-ray skull, blood tests	Generalized atrophy	Presenile dementia	n/a

Abbreviations: BNA-R = Behavioral Neurology Assessment; CT = computed tomography; DA = developmental age; DSDS = Dementia Scale for Down syndrome; EF = executive functioning; f/m = female/male; FSIQ = Full-Scale Intelligence Quotient; FXTAS = Fragile-X–associated tremor ataxia syndrome; LP = lumbar puncture; MMSE = Mini-Mental State Examination; MoCA = Montreal Cognitive Assessment; NPA = neuropsychological assessment; SRZ = the social competence rating scale for persons with an intellectual disability; WAIS = Wechsler Adult Intelligence Scale; WM = white matter; WRAT-2 = Wide-Range Achievement Test.

a Separate case reports of adults in children cohorts.

b Average age at first dementia encounter.

#### Etiology

The assumed etiology was specified in 9/23 studies that reported on dementia (39.1%). A clinical impression of AD was reported in 3 individuals of a Prader-Willi cohort and a separate case study, all women aged 40–58 years with the maternal uniparental disomy subtype and a history of psychosis, although authors did not clarify how this impression was substantiated.^e30,e40^ An 18-year-old man with Phelan-McDermid syndrome and late-onset psychiatric features and sleep disturbances was diagnosed with bipolar disorder and possible AD, which was substantiated by CSF concentrations of low Aβ, low total tau, and normal phosphorylated tau protein.^e38^ The AD diagnosis in neurofibromatosis type 1 were likely formal because these were retrieved by ICD-10 codes, but diagnostic details were not provided.^e17^ A cohort study in Williams syndrome reported on 3 cases of vascular dementia, with no further details.^e28^ Four men with fragile X syndrome^e12,e22,e25,e26^ met criteria for fragile X–associated tremor/ataxia syndrome (FXTAS) because of the onset of parkinsonism and dementia along with findings of elevated FMR1 mRNA levels^e22,e26^ and intranuclear inclusions characteristic of FXTAS pathology.^e25^ In 3 of these individuals, this was explained by a *size* mosaic form of fragile X syndrome, defined as those with both premutation and full mutation cells. The fourth man had a fully unmethylated, full mutation.^e25^ A probable diagnosis of a behavioral variant of frontotemporal dementia was reported in a 52-year-old man with a subclinical form of tuberous sclerosis complex,^e24^ based on behavioral changes, neuropsychological profile, and MRI findings.

#### Age at Onset

The identified individuals with dementia had a mean age at onset of 44.4 (range 16–71, median 40) years, of whom 21/49 (42.9%) had young-onset dementia (younger than 65 years). No association with young-onset dementia was found in neurofibromatosis type 1, in which the average age at *first dementia encounter* (see above) was 74.2 (range 65.2–83.2) years or in the mosaicism cases of fragile X syndrome with dementia onset in the sixth or seventh decade.

#### Behavioral and Psychological Symptoms

Reported behavioral and psychological symptoms of dementia included most often changes in mood (10/49, 20.4%), aggression (9/49, 18.4%), and obsessive-compulsive behavior (5/49, 10.2%) (eTable 7). In a Williams syndrome cohort, common symptoms of dementia were explicitly mentioned, including weight change, change in appetite, onset of or increase in physical aggression, and reduced quantity of speech.^e28^ A subgroup of patients with 22q11.2 deletion syndrome and significant intellectual decline displayed more symptoms of depressive and psychotic disorders than those without cognitive decline, memory and concentration problems, restlessness, sleep problems, and anhedonia.^e9^

#### Dementia Diagnosis

Diagnostic methods were rarely specified in cohort studies; in total, not for half of the reported individuals (n = 25/49, 51.0%). In the others, cognitive decline was substantiated by neuropsychological assessment (NPA) in most (n = 18/49, 36.7%). Language (n = 8) and memory (n = 7) were cognitive domains affected most often. In none of the cases with Phelan-McDermid (n = 4), NPA was reported, although authors presumed dementia or neurodegeneration. Additional medical examinations included the following: MRI (n = 12/49, 24.5%), blood tests (n = 3/49, 6.1%), lumbar puncture (n = 2/49, 4.1%), computed tomography (n = 2/49, 4.1%), autopsy (n = 2/49, 4.1%), PET (n 1/49, 2.0%), X-ray scan (1/49, 2.0%), and electroencephalography (n = 1/49, 2.0%).

### Comorbidity

#### Epilepsy

In a cohort of adults with neurofibromatosis type 1, epilepsy was more frequent in individuals with dementia.^e17^ In a fragile X cohort, lower intelligence was reported in 1 individual with increased seizure frequency at follow-up.^e27^ In a family with ring chromosome 20 syndrome, younger age at seizure onset (5 and 7 years) was associated with worse cognitive trajectories.^e14^ In 2 siblings with Phelan-McDermid syndrome, cognition declined progressively after adult-onset epilepsy and psychosis.^e18^ In a cohort of late-diagnosed adults with Dravet syndrome with and without *SCN1A* mutations (n = 22), cognitive decline was partially reversible after seizure control in 2 individuals (after as long as 60 years of drug-resistant epilepsy), with neuropathologic research ruling out neurodegeneration.^e7^

#### Movement Disorders

FXTAS with dementia was reported in all cases with mosaic fragile X mentioned earlier, Parkinson disease with cognitive decline in 4/44 (9.1%) male individuals with full fragile X mutations,^e33^ cerebellar atrophy and parkinsonism with cognitive decline in 9/9 (100%) individuals with Dravet syndrome,^e7^ and gait abnormalities with onset of psychiatric illness and functional regression in 7/38 (18.4%) individuals with Phelan-McDermid syndrome.^e19^

#### Psychotic Disorders

In 22q11.2 deletion syndrome, (a history of) psychotic episodes and schizophrenia were associated with cognitive decline in 5 partially overlapping cohorts^e2,e8,e9,e23,e39^ and in 6 separate cases.^e10^ In 4/5 cohorts, psychosis onset occurred in adolescence (mean cohort age 21.3 years), and cognitive decline preceded psychosis onset. In 1 Prader-Willi cohort, a history of psychosis was considered a primary risk factor of young-onset dementia,^e40^ with 1 patient meeting criteria for *at least mild dementia* after a psychotic episode. In 2 separate case reports of mosaic Turner syndrome with schizophrenia, neuroimaging indicated significant cerebral atrophy.^e3,e21^

#### Cardiovascular Disorders

For older individuals with neurofibromatosis type 1, there was no significant association between hypertension and dementia.^e17^ In the cohort with Williams syndrome,^e28^ abundant cardiovascular comorbidity was reported including hypertension (77%), congenital heart defects (45%), and a history of transient ischemic attacks (23%); however, the presence of cardiovascular comorbidity in the 3 identified cases with dementia was not specified.

#### Medication

One retrospective observational study in 22q11.2 deletion syndrome suggested positive correlates between selective serotonin reuptake inhibitors (SSRIs) and IQ measures, hippocampal volume, and cortical thickness.^e23^ A positive cognitive effect of the ASM valproate was suggested in Dravet syndrome (see earlier).^e7^

### Neuropsychological Assessment

Across all included studies, a variety of 44 different psychodiagnostic instruments were reported in 10 RGNDs and 28 studies (Table 2). Cognition was most often assessed with versions of the Wechsler Adult Intelligence Scale (in 13 studies), Wechsler Intelligence Scale for Children (in 8 studies), or the screener Mini-Mental State Examination (in 7 studies). Adaptive functioning was most often assessed with the Vineland Adaptive Behavior Scales (in 5 studies).

**Table 2:** Use of Psychodiagnostic Instruments in Rare Genetic Neurodevelopmental Disorders

RGND	Studies (N)	Cognitive functioning (in N studies)	Adaptive functioning (in N studies)	Other (in N studies)
22q11.2 deletion syndrome	8	WISC-III/IV/R (6) WAIS–III/IV (6) WPPSI (2) Conners CPT-2 or 3 (1) CVLT (1) DMR (1) GDS (1) GIT (1) IQCODE (1) PPVT (1) Visual span test (1) WCST (1) WIAT-II (1)	ABCL (2) VABS (2)	Pennsylvania emotion recognition test (1)
Fragile X syndrome	6	MMSE (4) WAIS-III/R (3) ACE-R (1) Color form sorting test (1) FAB (1) FRWT (1) GBT (1) LIPS (1) Merrill-Palmer test (1) Stanford-Binet test (1) WASI (1) WCST (1) WMS-III (1)	W-ADL (1)	n/a
Williams syndrome	4	WAIS-III/IV (2) BPVS (1) BSID-II (1) Buschke's SRT(1) EOWPVT (1) LIPS (1) Snodgrass' picture fragment completion (1) WISC-R (1) WPPSI (1)	VABS (1)	PPS-LD (1)
Prader-Willi syndrome	2	CAMDEX-DS (1) DSDS (1) Merill-Palmer test (1) Stanford-Binet (1)	SRZ (1) VABS (1)	n/a
Mbd5-associated neurodevelopmental disorder	2	WISC-III (1) MMSE (1) FAB (1) Verbal fluency animals (1)	VABS-Z (1)	SEO-R (1)
Ring chromosome 20 syndrome	2	WAIS (2)	n/a	n/a
Cardiofaciocutaneous syndrome	1	BNA-R (1) CDR (1) WRAT-2 (1)	n/a	n/a
Dravet syndrome	1	MMSE (1)	n/a	n/a
Phelan-McDermid syndrome	1	ITPA (1) Merill-Palmer test (1) WPPSI-R (1)	n/a	n/a
3q29 deletion syndrome	1	MMSE (1) MoCA (1)	n/a	n/a

Abbreviations: ACE = Addenbrooke's Cognitive Examination; BNA = Behavioral Neurologic Assessment; BPVS = British Picture Vocabulary Scale; BSID = Bayley Scales of Infant Development; CAMDEX-DS = Cambridge Examination for Mental Disorders of Older People with Down Syndrome and Others with ID; CDR = Clinical Dementia Rating Scale; Conners CPT = Continuous Performance Test; CVLT = California Verbal Learning Test; DMR = Dementia Questionnaire for Persons with Mental Retardation; DSDS = Dementia Scale for Down Syndrome; EOWPVT = Expressive One-Word Picture Vocabulary Test; FAB = frontal assessment battery; FRWT = Face Recognition of Warrington Test; GBT = Grober and Buschke Test; GDS = Gordon Diagnostic System; GIT = Groninger Intelligence Test; ITPA = Illinois Test of Psycholinguistic Abilities; IQCODE = Informant Questionnaire on Cognitive Decline in the Elderly; LIPS = Leiter International Performance Scale; MMSE = Mini-Mental State Examination; MoCA = Montreal Cognitive Assessment; PPS-LD = Present Psychiatric State–Learning Disabilities assessment; PPVT = Peabody Picture Vocabulary Test; RGND = rare genetic neurodevelopmental disorder; SEO-R = Dutch Scale for Emotional Development in people with ID; SRT = selective reminding task; SRZ = The Dutch Social Functioning Scale for ID; VABS = Vineland Adaptive Behavior Scale; W-ADL = Waisman Activities of Daily Living scale; WAIS = Wechsler Adult Intelligence Scale; WASI = Wechsler Abbreviated Scale of Intelligence; WCST = Wisconsin Card Sorting Test; WIAT = Wechsler Individual Achievement Test; WISC = Wechsler Intelligence Scale for Children; WMS-III = Wechsler Memory Scale; WRAT = Wide-Range Achievement Test.

### Quality Assessment

Results regarding study quality, as assessed by the Newcastle-Ottawa scale, are listed in eTable 3. Summarized, most cohort studies were rated to have some limitations in quality by missing stars in at least 1 criterion. The main information missing related to representativeness, mostly due to missing information on unexposure, nonresponse bias, or high percentages lost to follow-up.

## Discussion

This study provides the first systematic review of cognitive and adaptive trajectories of adults with RGNDs, focusing on dementia. With now more than 1,700 known ID-related genetic disorders,^26^ the current yield of 40 reports in 14 different RGNDs reveals a gap in understanding and reporting of cognitive aging in this population.

In total, dementia was reported in 49 individuals with 12 genetic syndromes and with various dementia etiologies, diagnostic certainties, and diagnostic methods. Given the rarity of the disorders, cohorts were often small in size and sampled from convenience sources such as consortia or care facilities, making prevalence rates unsuitable for generalization. An exception was one large national registry study of individuals with neurofibromatosis type 1, providing the most robust evidence on elevated hazard risks for all types of dementia and AD. This is probably due to neurofibromatosis type 1 being less rare, and with a relative mild neurocognitive phenotype facilitating dementia diagnosis, underlining a diagnostic care gap in more rare and severe RGNDs.

The presumed dementia etiology was specified in few articles. In Prader-Willi syndrome, the presumed young-onset AD in 4 female individuals is consistent with previously reported neuropathologic features. All these individuals had uniparental disomy, which has been associated with psychosis risk^27^ and may also increase susceptibility of young-onset AD in Prader-Willi syndrome. Another factor might be female sex because decreasing sex hormones during menopause have been associated with both schizophrenia spectrum disorders^28^ and AD^29^ The role of sex hormones might be particularly crucial in Prader-Willi syndrome because hypogonadism and early menopause are common.^30^ A possible case of AD in Phelan-McDermid syndrome fits the phenotype of regression in adolescents with Phelan-McDermid syndrome following psychiatric episodes^31^ and is the first to report CSF biomarkers indicative of AD. Of note are the reported clinical and neuropathologic findings of FXTAS in cases with mosaic or unmethylated full *FMR1* mutations in fragile X syndrome, considering that FXTAS was historically considered to be exclusive to the premutation carriers. While dementia in FXTAS is reported to share features with AD and APP-upregulation,^32^ both neuropathologic^33^ and neuropsychological^34^ FXTAS characteristics primarily resemble a white matter dementia. The psychiatric manifestations in a man with tuberous sclerosis complex (TSC) were reported to resemble the behavioral variant of frontotemporal dementia (bvFTD), consistent with recent neuropathologic findings of an aging-associated *TSC tauopathy*,^35,36^ in which the mTOR pathway hyperactivation and elevated phosphorylated tau aggregates are associated with premature neurodegeneration. Because psychiatric manifestations are part of TSC-associated neuropsychiatric disorders,^37^ this underlines the necessity of systematic monitoring of the behavioral adult phenotypes to identify new-onset disorders.

This review shows that dementia can have a young age at onset in individuals with some but not all RGNDs. The identified high prevalence rate of young-onset dementia in this review (42.9%) is very likely subject to publication bias but still noteworthy considering the global age-standardized prevalence of young-onset dementia (<1%). In a recent review on causes of young-onset dementia,^38^ several rare genetic disorders were identified, illustrating that increased understanding of young-onset dementia from a genetic point of view is pivotal. The current findings suggest that in addition to DS, other genetic neurodevelopmental disorders predispose to young-onset dementia as well. This is concordant with accumulating evidence of overlapping genes, proteins, and pathways involved in both abnormal neurodevelopment and neurodegeneration,^39,40^ providing a context of a neurodevelopmental-degenerative continuum rather than dichotomous separation.

Common behavioral and psychological dementia symptoms in RGNDs such as mood changes, aggression, and obsessive-compulsive behaviors are also well-recognized in the general population with ID. Behavioral and psychological symptoms vary between ID levels and are considered to be relevant dementia indicators in severe/profound ID particularly^41^ because changes in cognition are more difficult to substantiate.^20^ Considering the underrepresentation of severe/profound ID in this review, more research into dementia symptoms would particularly benefit this vulnerable subgroup.

Diagnostic methods were in accordance with current guidelines in 10 studies (20.4%), combining neuropsychological assessment and neuroimaging, although even this small number may be an overestimation because diagnostic methods for individuals in cohorts were rarely reported. In few occasions, other investigations to increase diagnostic certainty were reported, such as lumbar puncture (n = 2/49, 4.1%), PET (n = 1/49, 2.0%), or postmortem neuropathologic research (n = 1/49, 2.0%). In our clinical experience, these limited diagnostics reflect difficulties in performing lumbar puncture and imaging procedures, often necessitating sedation or anesthesia in a population in which anxiety disorders and medical phobias are common. It is notable that only 1 person with mild cognitive impairment was reported, raising the question whether early cognitive decline is underdiagnosed or misinterpreted as part of natural aging trajectories. Considering the impact of a dementia diagnosis and prognosis, and the limitations of neuropsychological instruments in ID (see *Neuropsychological assessment*), individuals and families should be offered additional investigations and postmortem neuropathologic confirmation to increase diagnostic accuracy.^42^ Very promising for this patient population are the rapid developments of blood-based biomarkers for neurodegeneration and dementia, which are less invasive, less costly,^43^ and recently recommended for prescreening purposes.^44^

**Figure 3:**
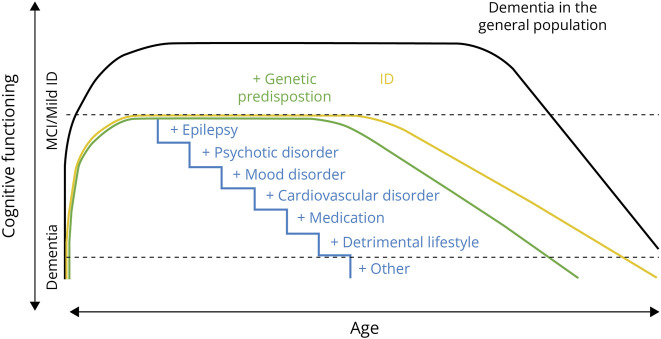
Aging Trajectories of Cognitive Functioning ID = intellectual disability, MCI = mild cognitive impairment.

In various RGNDs, associations between epilepsy characteristics and progressive cognitive decline were reported. Neuropsychological deficits are almost always reported in epilepsy, but the differential effects of seizures and the underlying neuronal abnormalities are unclear. A growing body of literature shows relations between epilepsy, mTOR pathways, tauopathy,^35,36,45^ and neuronal activity–dependent synaptic release of dementia biomarkers,^46,47^ suggesting a link between neurodevelopmental disorders, epilepsy, and neurodegeneration. A developmental and epileptic encephalopathy is sometimes considered a (partly) reversible dementia, exemplified by the cohort with late-diagnosed Dravet adults in which some individuals improved cognitively even after decades of treatment resistance. This underlines the importance of epilepsy screening and adequate treatment with ASM. To integrate the epilepsy factors and other potential *second hits* in RGNDs, an adaption of the previously proposed *chronic accumulation model*^16^ is depicted in Figure 3.

Coexistence of cognitive decline and movement disorders in RGNDs are in line with previous findings in RGNDs,^48^ reporting cognitive decline or dementia in 16.6% of those with signs of parkinsonism. Our results show how neurodegeneration in some RGNDs may result in shared cognitive and motor symptomatology, which corresponds with secondary dementias known to certain neurodegenerative diseases, such as Parkinson disease, amyotrophic lateral sclerosis, and Huntington disease. This is important because some RGNDs are genetically predisposed to an elevated risk of parkinsonism, such as 22q11.2 deletion syndrome and those with pathogenic *RAB39b* variants.^48^ Collaboration between neurologists and RGND experts will aid timely recognition and treatment of all neurodegenerative disorders, improving overall care.

The abundance of reported associations between psychotic disorders and dementia/cognitive decline is not surprising because psychotic (*neuropsychiatric*) symptoms can occur in dementia, and cognitive symptoms and cognitive decline are a core symptom of schizophrenia. In the past, schizophrenia has long been considered a neurodegenerative disease (*dementia praecox*). Contrary to this neurodegenerative phenotype is the more modern neurodevelopmental model of schizophrenia, proposing that this abnormal neurodevelopmental trajectory is already established in gestation, long before clinical symptoms occur in early adult life. Our findings fit within a neurodevelopmental-degenerative-continuum^17^ that proposes schizophrenia is a disorder with vulnerability throughout different life stages, observed as more *typical* schizophrenia trajectories with onset in adolescence in 22q11.2 deletion syndrome and as a chronic and progressive decline in older adults with RGNDs. This emphasizes that dementia should not be excluded from differential diagnostics in RGNDs suffering psychotic comorbidity.

Vascular dementia in Williams syndrome may be an example of how cardiovascular and lifestyle-related risk factors might predispose or protect individuals from specific dementia etiologies such as vascular dementia or AD. Treatment of hypertension and obesity along with diet and physical exercise should remain an area of improvement in all IDs and specifically in RGNDs predisposed to hyperphagia and cardiovascular risk.

The possible beneficial cognitive effects of SSRIs in 22q11.2 deletion syndrome and ASM in Dravet syndrome emphasize the importance of recognizing and treating states of pseudodementia caused by other disorders such as epilepsy or mood/anxiety disorders. A recent meta-analysis showed associations between some first-generation ASMs and greater dementia risk; however, verification by more prospective studies is warranted.^49^

**Figure 4:**
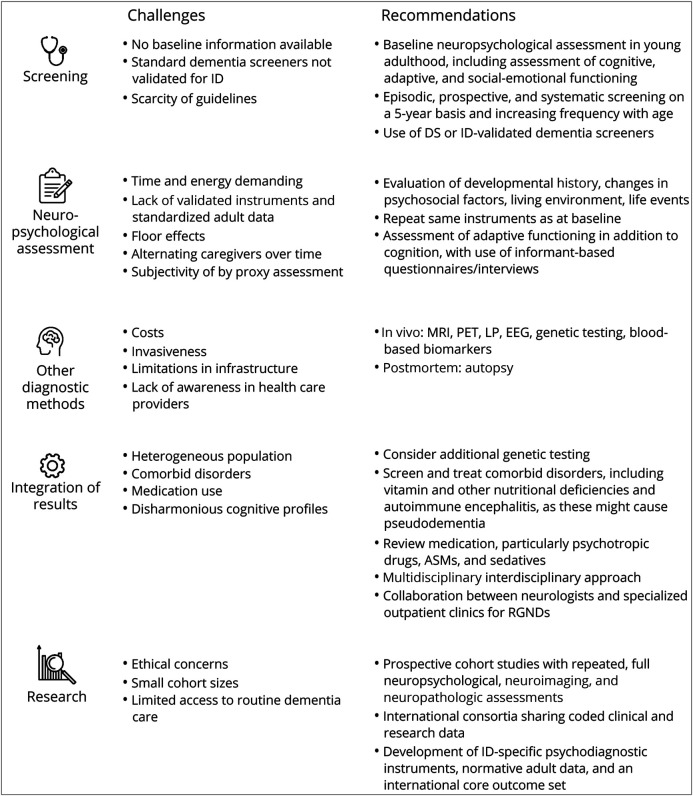
Challenges and Recommendations for Dementia Diagnostics and Research in Rare Genetic Neurodevelopmental Disorders ASM = antiseizure medication; DS = Down syndrome; ID = intellectual disability; LP = lumbar puncture; RGND = rare genetic neurodevelopmental disorder.

A variety of 44 different psychodiagnostic instruments was reported with little uniformity within and between RGNDs, reflecting the challenges clinicians face in selecting appropriate instruments in this population (Figure 4) and the need for a core outcome set for ID and/or RGNDs. Age-appropriate instruments might result in floor effects,^19^ while pediatric instruments lack normative data for adults. Recent reviews confirmed that only approximately half of applied instruments in ID are valid and reliable.^50,51^ This review also shows that dementia screening was often initiated only when concerns emerged.

Increasingly, recommendations are available for monitoring of cognitive functioning in RGNDs such as for 22q11.2 deletion syndrome, tuberous sclerosis complex,^37^ and DS.^2^ For the 1,700 RGNDs awaiting specific guidelines and for ID of unknown etiology, recommendations are proposed and summarized in Figure 4. Given that both dementia and most RGNDs are characterized by impairments in cognitive and adaptive functioning, longitudinal monitoring is necessary to substantiate decline from a previous level of functioning in individuals with RGNDs. Ideally, a baseline measurement should capture peak functioning in the adult life of an individual before onset of decline, typically from the age of 25 years. However, based on the very young onset of dementia in some of the identified RGNDs and even reports of decline in the second decade, we advise a broad, neuropsychological baseline measurement in all individuals with RGNDs starting in the beginning of adulthood and ideally before the transition to other daytime or living environments at approximately 18 years of age. From then on, this should be followed by a minimum of 5-yearly screening (increasing with age).^2^ These recommendations might raise concerns about validity and time consumption, but the DLD, DSQIID, and CAMDEX-DS are validated in the population with ID with a limited burden for informants.^50^ More validation and systematic evaluation are necessary to develop an international core outcome set for longitudinal direct assessment.

Strengths of this systematic review include the comprehensive search strategy, the extensive data collection, the study quality assessment, and a multidisciplinary research team. Limitations include the retrospective nature and convenience sampling of most studies, the likelihood of publication bias, the fact that study populations were often small due to the rarity of the disorders, and that controls were often lacking. This is reflected by the limitations in study quality in some studies because samples were not always representative. Individuals of older age or with moderate to severe levels of ID were underreported, reflecting a care and research gap.

This review reveals diagnostic and reporting gaps in dementia and cognitive/adaptive trajectories in adults with RGNDs. Our findings underline that systematic longitudinal follow-up of cognitive and adaptive functioning, using validated instruments, from young adulthood is justified to improve diagnosis and anticipatory care. Future research is required to further investigate the neurodevelopmental-neurodegenerative continuum in RGNDs, including the prevalence, phenomenology, and pathophysiology of (young-onset) dementia. More insights into genetic and other risk factors will provide a basis for targeted screening, diagnostics, monitoring and guidelines, optimizing personalized care for this vulnerable patient population.

## Appendix Authors

**Table:**

Name	Location	Contribution
Hadassa Kwetsie, MSc	Emma's Children's Hospital, University of Amsterdam; Advisium, 's Heeren Loo Zorggroep, Amersfoort, The Netherlands	Drafting/revision of the article for content, including medical writing for content; major role in the acquisition of data; and analysis or interpretation of data
Malu van Schaijk, MSc	Department on Aging, Netherlands Institute of Mental Health and Addiction (Trimbos Institute), Utrecht, The Netherlands	Drafting/revision of the article for content, including medical writing for content; major role in the acquisition of data; study concept or design; and analysis or interpretation of data
Sven Van Der Lee, MD, PhD	Alzheimer Center Amsterdam, Amsterdam University Medical Center; Section Genomics of Neurodegenerative Diseases and Aging, Department of Human Genetics Amsterdam UMC, The Netherlands	Drafting/revision of the article for content, including medical writing for content; study concept or design; and analysis or interpretation of data
Dederieke Maes-Festen, MD, PhD	Intellectual Disability Medicine, Department of General Practice, Erasmus MC, University Medical Center Rotterdam, The Netherlands	Drafting/revision of the article for content, including medical writing for content; analysis or interpretation of data
Leontine W. Ten Hoopen, MD	ENCORE Expertise Center for Neurocognitive Disorders and Department of Pediatric Neurology, Sophia Children's hospital, Erasmus MC University Medical Center Rotterdam; Erasmus School of Health Policy & Management, Erasmus University Rotterdam, The Netherlands	Drafting/revision of the article for content, including medical writing for content; major role in the acquisition of data; and analysis or interpretation of data
Mieke M. van Haelst, MD, PhD	Department of Clinical Genetics; Department of Human Genetics, Amsterdam UMC, University of Amsterdam; Emma Center for Personalized Medicine, Amsterdam University Medical Centers, The Netherlands	Drafting/revision of the article for content, including medical writing for content; analysis or interpretation of data
Michael Coesmans, MD, PhD	Department of Psychiatry, Erasmus MC University Medical Center, Rotterdam, The Netherlands	Drafting/revision of the article for content, including medical writing for content; analysis or interpretation of data
Esther Van Den Berg, PhD	Department of Neurology and Alzheimer Center Erasmus MC, Erasmus MC University Medical Center, Rotterdam, The Netherlands	Drafting/revision of the article for content, including medical writing for content
Marie Claire Y. De Wit, MD, PhD	ENCORE expertise center for neurocognitive disorders and department of Pediatric Neurology, Sophia Children's hospital, Erasmus MC University Medical Center Rotterdam, The Netherlands	Drafting/revision of the article for content, including medical writing for content
Yolande Pijnenburg, MD, PhD	Alzheimer Center Amsterdam, Amsterdam University Medical Center; Amsterdam Neuroscience, Neurodegeneration, Amsterdam, The Netherlands	Drafting/revision of the article for content, including medical writing for content
Eleonora Aronica, MD, PhD	Amsterdam UMC, University of Amsterdam, Department of (Neuro)Pathology, Amsterdam Neuroscience; Stichting Epilepsie Instellingen Nederland (SEIN), Heemstede, The Netherlands	Drafting/revision of the article for content, including medical writing for content
Erik Boot, MD, PhD	Advisium, 's Heeren Loo Zorggroep, Amersfoort; The Dalglish Family 22q Clinic, University Health Network, Toronto, Canada; Department of Psychiatry and Neuropsychology, Maastricht University, Maastricht university, The Netherlands	Drafting/revision of the article for content, including medical writing for content; analysis or interpretation of data
Agnies M. Van Eeghen, MD, PhD	Emma's Children's Hospital, University of Amsterdam; Advisium, 's Heeren Loo Zorggroep, Amersfoort; Emma Center for Personalized Medicine, Amsterdam University Medical Centers, The Netherlands	Drafting/revision of the article for content, including medical writing for content; major role in the acquisition of data; study concept or design; and analysis or interpretation of data

## Glossary

AD: Alzheimer disease
ASM: antiseizure medication
DS: Down syndrome
FSIQ: full-scale IQ
FXTAS: fragile X–associated tremor/ataxia syndrome
ID: intellectual disability
NPA: neuropsychological assessment
RGNDs: rare genetic neurodevelopmental disorders
TSC: tuberous sclerosis complex

## References

[1] Nguengang Wakap S, Lambert DM, Olry A, et al. Estimating cumulative point prevalence of rare diseases: analysis of the Orphanet database. Eur J Hum Genet. 2020;28(2):165-173. doi:10.1038/s41431-019-0508-0 31527858 PMC6974615

[2] Tsou AY, Bulova P, Capone G, et al. Global Down Syndrome Foundation medical care guidelines for adults with Down syndrome workgroup. Medical care of adults with Down syndrome: a clinical guideline. JAMA. 2020 Oct 20;324(15):1543-1556. doi:10.1001/jama.2020.17024 33079159

[3] Janicki MP, Dalton AJ. Prevalence of dementia and impact on intellectual disability services. Ment Retard. 2000 Jun;38(3):276-88 doi: 10.1352/0047-6765(2000)038<0276:PODAIO>2.0.CO;2. 10900935

[4] Van Schrojenstein Lantman-de Valk HMJ, Van Den Akker M, Maaskant MA, et al. Prevalence and incidence of health problems in people with intellectual disability. J Intellect Disabil Res. 1997;41(1):42-51. doi:10.1111/j.1365-2788.1997.tb00675.x 9089458

[5] Zigman WB, Schupf N, Devenny DA, et al. Incidence and prevalence of dementia in elderly adults with mental retardation without Down syndrome. Am J Ment Retard. 2004;109(2):126-141. doi:10.1352/0895-8017(2004)109<126:IAPODI>2.0.CO;2 15000676

[6] Cooper SA. High prevalence of dementia among people with learning disabilities not attributable to down's syndrome. Psychol Med. 1997;27(3):609-616. doi:10.1017/s0033291796004655 9153681

[7] Hewitt KE, Fenner ME, Torpy D. Cognitive and behavioural profiles of the elderly mentally handicapped. J Ment Defic Res. 1986;30(Pt 3):217-225. doi:10.1111/j.1365-2788.1986.tb01316.x 3783659

[8] Strydom A, Chan T, King M, Hassiotis A, Livingston G. Incidence of dementia in older adults with intellectual disabilities. Res Dev Disabil. 2013;34(6):1881-1885. doi:10.1016/j.ridd.2013.02.021 23578903

[9] Strydom A, Hassiotis A, King M, Livingston G. The relationship of dementia prevalence in older adults with intellectual disability (ID) to age and severity of ID. Psychol Med. 2009;39(1):13-21. doi:10.1017/S0033291708003334 18410700

[10] Strydom A, Livingston G, King M, Hassiotis A. Prevalence of dementia in intellectual disability using different diagnostic criteria. Br J Psychiatry. 2007;191:150-157. doi:10.1192/bjp.bp.106.028845 17666500

[11] Takenoshita S, Terada S, Kuwano R, et al. Prevalence of dementia in people with intellectual disabilities: cross-sectional study. Int J Geriatr Psychiatry. 2020;35(4):414-422. doi:10.1002/gps.5258 31894597

[12] Silverman WP, Zigman WB, Krinsky-McHale SJ, Ryan R, Schupf N. Intellectual disability, mild cognitive impairment, and risk for dementia. J Policy Pract Intellect Disabil. 2013;10(3):245-251. doi:10.1111/jppi.12042 PMC3834861 24273589

[13] Moss S, Patel P. Psychiatric symptoms associated with dementia in older people with learning disability. Br J Psychiatry. 1995;167(5):663-667. doi:10.1192/bjp.167.5.663 8564325

[14] Shooshtari S, Martens PJ, Burchill CA, Dik N, Naghipur S. Prevalence of depression and dementia among adults with developmental disabilities in Manitoba, Canada. Int J Fam Med. 2011;2011:319574. doi:10.1155/2011/319574 PMC3263837 22295184

[15] Stern Y. Cognitive reserve in ageing and Alzheimer's disease. Lancet Neurol. 2012;11(11):1006-1012. doi:10.1016/S1474-4422(12)70191-6 23079557 PMC3507991

[16] Breuer LEM, Boon P, Bergmans JWM, et al. Cognitive deterioration in adult epilepsy: does accelerated cognitive ageing exist? Neurosci biobehavioral Rev. 2016;64:1-11. doi:10.1016/j.neubiorev.2016.02.004 26900650

[17] Stone WS, Phillips MR, Yang LH, Kegeles LS, Susser ES, Lieberman JA. Neurodegenerative model of schizophrenia: growing evidence to support a revisit. Schizophr Res. 2022;243:154-162. doi:10.1016/j.schres.2022.03.004 35344853 PMC9189010

[18] Douglas KM, Gallagher P, Robinson LJ, et al. Prevalence of cognitive impairment in major depression and bipolar disorder. Bipolar Disord. 2018;20(3):260-274. doi:10.1111/bdi.12602 29345037

[19] Elliott-King J, Shaw S, Bandelow S, Devshi R, Kassam S, Hogervorst E. A critical literature review of the effectiveness of various instruments in the diagnosis of dementia in adults with intellectual disabilities. Alzheimers Dement (Amst). 2016;4:126-148. doi:10.1016/j.dadm.2016.06.002 27752536 PMC5061450

[20] Page MJ, McKenzie JE, Bossuyt PM, et al. The PRISMA 2020 statement: an updated guideline for reporting systematic reviews. Rev Esp Cardiol (Engl Ed). 2021;74(9):790-799. doi:10.1016/j.rec.2021.07.010 34446261

[21] Association AP. APA Dictionary of Psychology, 2nd ed; 2015.

[22] Orphanet. Accessed February 7, 2022. www.orpha.net.

[23] Ouzzani M, Hammady H, Fedorowicz Z, Elmagarmid A. Rayyan-a web and mobile app for systematic reviews. Syst Rev. 2016;5(1):210. doi:10.1186/s13643-016-0384-4 27919275 PMC5139140

[24] GA Wells BS, O'Connell D, Peterson J, Welch V, Losos M, Tugwell P. The Newcastle-Ottawa Scale (NOS) for assessing the quality of nonrandomised studies in meta-analyses [cited 2023]. ohri.ca/programs/clinical_epidemiology/oxford.asp

[25] Cochrane. Accessed December 6, 2022. cccrg.cochrane.org

[26] Maia N, Nabais Sa MJ, Melo-Pires M, de Brouwer APM, Jorge P. Intellectual disability genomics: current state, pitfalls and future challenges. BMC Genomics. 2021;22(1):909. doi:10.1186/s12864-021-08227-4 34930158 PMC8686650

[27] Yang L, Zhan GD, Ding JJ, et al. Psychiatric illness and intellectual disability in the Prader-Willi syndrome with different molecular defects—a meta analysis. PLoS One. 2013;8(8):e72640. doi:10.1371/journal.pone.0072640 23967326 PMC3743792

[28] Sommer IE, Brand BA, Gangadin S, Tanskanen A, Tiihonen J, Taipale H. Women with schizophrenia-spectrum disorders after menopause: a vulnerable group for relapse. Schizophr Bull. 2023;49(1):136-143. doi:10.1093/schbul/sbac139 36198044 PMC9810004

[29] Radaghdam S, Karamad V, Nourazarian A, Shademan B, Khaki-Khatibi F, Nikanfar M. Molecular mechanisms of sex hormones in the development and progression of Alzheimer's disease. Neurosci Lett. 2021;764:136221. doi:10.1016/j.neulet.2021.136221 34500000

[30] Pellikaan K, Ben Brahim Y, Rosenberg AGW, et al. Hypogonadism in women with prader-willi syndrome-clinical recommendations based on a Dutch cohort study, review of the literature and an international expert panel discussion. J Clin Med. 2021;10(24):5781. doi:10.3390/jcm10245781 34945077 PMC8707541

[31] Kolevzon A, Delaby E, Berry-Kravis E, Buxbaum JD, Betancur C. Neuropsychiatric decompensation in adolescents and adults with Phelan-McDermid syndrome: a systematic review of the literature. Mol Autism. 2019;10:50. doi:10.1186/s13229-019-0291-3 31879555 PMC6930682

[32] Sokol DK, Maloney B, Long JM, Ray B, Lahiri DK. Autism, Alzheimer disease, and fragile X: APP, FMRP, and mGluR5 are molecular links. Neurology. 2011;76(15):1344-1352. doi:10.1212/WNL.0b013e3182166dc7 21482951 PMC3090060

[33] Filley CM, Brown MS, Onderko K, et al. White matter disease and cognitive impairment in FMR1 premutation carriers. Neurology. 2015;84(21):2146-2152. doi:10.1212/WNL.0000000000001612 25925982 PMC4451047

[34] Grigsby J, Cornish K, Hocking D, et al. The cognitive neuropsychological phenotype of carriers of the FMR1 premutation. J Neurodev Disord. 2014;6(1):28. doi:10.1186/1866-1955-6-28 25136377 PMC4135346

[35] Hwang JHL, Perloff OS, Gaus SE, et al. Tuberous sclerosis complex is associated with a novel human tauopathy. Acta Neuropathologica. 2023;145(1):1-12. doi:10.1007/s00401-022-02521-5 36469115 PMC10244026

[36] Liu AJ, Lusk JB, Ervin J, Burke J, O'Brien R, Wang SHJ. Tuberous sclerosis complex is a novel, amyloid-independent tauopathy associated with elevated phosphorylated 3R/4R tau aggregation. Acta Neuropathol Commun. 2022;10(1):27. doi:10.1186/s40478-022-01330-x 35241183 PMC8896101

[37] de Vries PJ, Heunis TM, Vanclooster S, et al. International consensus recommendations for the identification and treatment of tuberous sclerosis complex-associated neuropsychiatric disorders (TAND). J Neurodev Disord. 2023;15(1):32. doi:10.1186/s11689-023-09500-1 37710171 PMC10503032

[38] van de Veen D, Bakker C, Peetoom K, et al. An integrative literature review on the nomenclature and definition of dementia at a young age. J Alzheimers Dis. 2021;83(4):1891-1916. doi:10.3233/JAD-210458 34487041 PMC8609678

[39] Hickman RA, O'Shea SA, Mehler MF, Chung WK. Neurogenetic disorders across the lifespan: from aberrant development to degeneration. Nat Rev Neurol. 2022;18(2):117-124. doi:10.1038/s41582-021-00595-5 34987232 PMC10132523

[40] Schor NF, Bianchi DW. Neurodevelopmental clues to neurodegeneration. Pediatr Neurol. 2021;123:67-76. doi:10.1016/j.pediatrneurol.2021.07.012 34399111 PMC10040214

[41] Wissing MBG, Ulgiati AM, Hobbelen JSM, De Deyn PP, Waninge A, Dekker AD. The neglected puzzle of dementia in people with severe/profound intellectual disabilities: a systematic literature review of observable symptoms. J Appl Res Intellect Disabil. 2022;35(1):24-45. doi:10.1111/jar.12920 34219327 PMC9292142

[42] McKhann GM, Knopman DS, Chertkow H, et al. The diagnosis of dementia due to Alzheimer's disease: recommendations from the National Institute on Aging-Alzheimer's Association workgroups on diagnostic guidelines for Alzheimer's disease. Alzheimers Dement. 2011;7(3):263-269. doi:10.1016/j.jalz.2011.03.005 21514250 PMC3312024

[43] Mattke S, Cho SK, Bittner T, Hlavka J, Hanson M. Blood-based biomarkers for Alzheimer's pathology and the diagnostic process for a disease-modifying treatment: projecting the impact on the cost and wait times. Alzheimers Dement (Amst). 2020;12(1):e12081. doi:10.1002/dad2.12081 32832590 PMC7434228

[44] Teunissen CE, Verberk IMW, Thijssen EH, et al. Blood-based biomarkers for Alzheimer's disease: towards clinical implementation. Lancet Neurol. 2022;21(1):66-77. doi:10.1016/S1474-4422(21)00361-6 34838239

[45] Aronica E, Specchio N, Luinenburg MJ, Curatolo P. Epileptogenesis in tuberous sclerosis complex-related developmental and epileptic encephalopathy. Brain. 2023;146(7):2694-2710. doi:10.1093/brain/awad048 36806388 PMC10316778

[46] Ismael S, Sindi G, Colvin RA, Lee D. Activity-dependent release of phosphorylated human tau from Drosophila neurons in primary culture. J Biol Chem. 2021;297(4):101108. doi:10.1016/j.jbc.2021.101108 34473990 PMC8455371

[47] Yamada K, Iwatsubo T. Extracellular α-synuclein levels are regulated by neuronal activity. Mol Neurodegener. 2018;13(1):9. doi:10.1186/s13024-018-0241-0 29467003 PMC5822605

[48] von Scheibler ENMM, van Eeghen AM, de Koning TJ, et al. Parkinsonism in genetic neurodevelopmental disorders: a systematic review. Mov Disord Clin Pract. 2022;10(1):17-31. doi:10.1002/mdc3.13577 36699000 PMC9847320

[49] Zhang L, Jiang HY, Liu WJ. Anti-seizure medication exposure and the risk of dementia: a meta-analysis of observational studies. Front Neurol. 2023;14:1133816. doi:10.3389/fneur.2023.1133816 37034066 PMC10073491

[50] Zeilinger EL, Zrnic Novakovic I, Komenda S, et al. Informant-based assessment instruments for dementia in people with intellectual disability: a systematic review and standardised evaluation. Res Dev Disabil. 2022;121:104148. doi:10.1016/j.ridd.2021.104148 34954669

[51] McKenzie K, Metcalfe D, Murray G. A review of measures used in the screening, assessment and diagnosis of dementia in people with an intellectual disability. J Appl Res Intellect Disabil. 2018;31(5):725-742. doi:10.1111/jar.12441 29424088

[R52] eReferences are available as supplemental digital content.

